# Facilitators and challenges in collaboration between public health units and faith-based organizations to promote COVID-19 vaccine confidence in Ontario

**DOI:** 10.1186/s12939-024-02326-w

**Published:** 2024-11-28

**Authors:** Kadidiatou Kadio, Denessia Blake-Hepburn, Melodie Yunju Song, Anna Karbasi, Elizabeth Estey Noad, Samiya Abdi, Nazia Peer, Shaza A. Fadel, Sara Allin, Anushka Ataullahjan, Erica Di Ruggiero

**Affiliations:** 1 Centre for Global Health, Dalla Lana School of Public Health, University of Toronto, 155 College St Room 500, Toronto, ON Canada; 2 Social and Behavioural Health Sciences Division, Dalla Lana School of Public Health, University of Toronto, Toronto, Canada; 3 Institute of Health Policy, Management, and Evaluation, Dalla Lana School of Public Health, University of Toronto, Toronto, Canada; 4 Centre for Vaccine Preventable Diseases, Dalla Lana School of Public Health, University of Toronto, Toronto, Canada; 5 North American Observatory on Health Systems and Policy (NAO), Dalla Lana School of Public Health, University of Toronto, Toronto, Canada; 6 Clinical Public Health Division, Dalla Lana School of Public Health, University of Toronto, Toronto, Canada; 7 School of Health Studies, Faculty of Health Sciences, Western University, London, Canada; 8 Institut de Recherche en Science de la Santé du Centre National de la Recherche Scientifique et Technologique (IRSS/CNRST), Ouagadougou, Burkina Faso; 9 Peel Public Health, Toronto, Canada

**Keywords:** Community engagement process, Faith-based organizations, COVID-19 vaccine, Public health partnerships, Facilitators and barriers factors, Populations made structurally vulnerable

## Abstract

**Background:**

Equitable access to vaccination remains a concern, particularly among population groups made structurally vulnerable. These population groups reflect the diversity of communities that are confronted with structural barriers caused by systemic racism and oppression and result in them experiencing suffer disadvantage and discrimination based on citizenship, race, ethnicity, ancestry, religion, spiritual beliefs, and/or gender identity. In Canada, Ontario public health units (PHUs) engage with faith-based organizations (FBOs) to improve vaccine confidence among populations made structurally vulnerable. This study explores the factors that facilitate and hinder engagement in the implementation of vaccine confidence promoting interventions, and challenges associated with working with FBOs.

**Methods:**

In-depth interviews were conducted with 18 of the 34 Ontario PHUs who expressed an interest. Braun and Clarke’s “experiential” approach was used to explore the realities of PHUs’ contextual experiences and perspectives.

**Results:**

The results showed that receptivity and openness of PHUs to learn from FBOs, previous experience working with religious communities and FBOs, ongoing relations based on respect of different beliefs and opinions on the vaccines, leveraging the support of trusted faith leaders among communities and communications strategy adapted and sensitive to the needs of the community was facilitators to community involvement in the prevention and control of COVID-19. On the other hand, factors both internal and external to the PHUs have often posed challenges to collaboration with the FBOs. Internal factors include low operational capacity of PHU like insufficient human and financial resources, weak analytical capacity, ambiguity in the roles and responsibilities of the different actors. Some external challenges issues were related to the provincial level and the Ministry of Health, while others were related to FBOs. For example, faith-based and collective beliefs promoting vaccine hesitancy have resulted in resistance from some religious communities when PHUs have reached out to collaborate.

**Conclusions:**

Engaging with faith-based communities is an ongoing process that requires time, flexibility, and patience, but it is necessary to improve vaccine confidence and equity access among population groups made structurally vulnerable. Lessons learned from this research can guide the implementation of future vaccination programs.

**Supplementary Information:**

The online version contains supplementary material available at 10.1186/s12939-024-02326-w.

## Introduction

COVID-19 vaccinations emerged in late 2020 as one of the priority public health strategies to control the COVID-19 pandemic. Notwithstanding the benefits of vaccination, the World Health Organization declared vaccine hesitancy, “the delay in acceptance or refusal of vaccination despite availability of vaccination services”, as a serious global threat [1–3]. Some critics point out that the term “vaccine hesitancy” does not take into account the range of causes of suboptimal vaccination and does not place enough emphasis on the social determinants of vaccination, potentially obscuring equity issues, and suggest the term “undervaccinated” to include people who are unvaccinated or partially vaccinated for whatever reason [4]. Its public health implications are far-reaching, leading to further disparities of receiving the benefits of vaccination [1, 2]. However, equitable access to vaccination remains a concern, particularly among population groups made structurally vulnerable [3, 5].

Several terms are used in the literature to designate different population groups: equity-seeking, equity-deserving, equity-denied, ethnic and racial groups, hard-to-reach populations, priority group, vulnerable or marginalized groups, to name a few [6, 7]. In this article we use the concept of structural vulnerability to focus on “an individual’s or a population group’s condition of being at risk for negative health outcomes through their interface with socioeconomic, political and cultural/normative hierarchies” [8]. Thus, the term group made structurally vulnerable reflects the diversity of communities that are confronted with structural barriers that cause them to experience systemic racism and oppression, suffer disadvantage and discrimination based on citizenship, race, ethnicity, ancestry, religion, spiritual beliefs, and/or gender identity.

COVID-19 disproportionately affected groups made structurally vulnerable [9, 10], whose vaccination coverage was below average [5, 11]. In Canada in 2022, the prevalence of COVID-19 ‘vaccine unwillingness’ was higher in non-white individuals (21.7%) compared with White individuals (14.8%) [12]. One of the few studies to analyze such issues in more detail found that African/Caribbean and Black (57.1%), indigenous (65.1%) and multiracial (77.8%) people were less likely to be vaccinated than white people (80.9%) [13]. This disparity is due to the fact that the trust in vaccines and the systems that deliver them has been particularly undermined by historical and contemporary contexts of systemic racism, marginalization, and oppression faced by Indigenous, black, and other racialized groups in Canada and other countries [5, 14–17]. Contemporary experiences must therefore be placed in the context of a history of medical abuse targeting Black, Indigenous and other oppressed communities [18–20].

Various strategies have been used to promote vaccine confidence among structurally vulnerable groups, such as community engagement [21, 22], which is one of the global guidelines for responding to the COVID-19 pandemic [23]. Community engagement refers to a continuum of actions that involve communities in decision making for planning, design, governance, and service delivery [24, 25]. Although not considered full engagement, it can simply involve providing information to partners to help understand a problem; however, the level of engagement can increase to include obtaining community feedback on options and decisions, regular interactions throughout the project cycle, or even collaboration and shared decision-making [24, 26–28].

Community engagement is considered essential for improving health equity among structurally vulnerable communities because it can positively influence action on the socio-structural determinants of health [29]. Ontario’s public health standards emphasize community engagement including in relation to health equity, which is a foundational standard [30].

During the pandemic, Ontario’s High Priority Communities Strategy (HPCS) was implemented to foster partnerships between public health agencies (PHAs) and faith-based and community organizations to overcome barriers to vaccine uptake and promote health equity [31]. In Ontario, Public Health Unit (PHU) is a health agency that provide health promotion and disease prevention programs. There are currently 34 PHUs and each has its own geographical boundaries. PHUs engagement with faith-based organizations (FBOs) can contribute to the achievement of equity goals by jointly implementing the principles of inclusion, flexibility, and promoting community trust in vaccines [32–34]. FBOs are defined as “entities whose organizational control, religious expression, and program implementation are tied to values and beliefs belonging to specific religious identities” (Bielefeld et Cleveland 2013). Their involvement is critical because they have the cultural competencies and relational capital and trust to foster open dialogue about vaccines [35, 36]. Studies conducted among communities made structurally vulnerable have shown that collaborations with FBOs help build confidence in the public health systems, increase vaccine acceptance rates and promote vaccine uptake [36, 37], and dispel myths and misconceptions about vaccines [15, 38].

However, little is known about the engagement processes that help or hinder the implementation of collaborative interventions between PHAs and FBOs. Studies have shown that FBO engagement with public health agencies contributes to improved vaccine coverage [33, 36, 39], but very little research has focused on the engagement process and the experiences of those involved, on the facilitators and barriers of PHU-FBO collaboration. By addressing these gaps, researchers and policymakers can strengthen these collaborations/partnerships that effectively enhance vaccine confidence within communities made structurally vulnerable. This study aimed to analyze Ontario public health partnerships with FBOs to improve vaccine confidence among populations made structurally vulnerable; explore the factors that facilitate and hinder engagement in the implementation of vaccine confidence promoting interventions, and challenges associated with working with FBOs.

## Methodology

This research is part of a larger mixed-methods study that aims to analyze Ontario PHUs’ partnerships with FBOs to improve vaccine confidence among populations made structurally vulnerable. This study aims to explore the factors that facilitate and hinder PHUs’ engagement with FBOs to implement vaccine confidence interventions, as well as the challenges associated with such collaborations.

### Study setting

The study was conducted in Ontario, which is home to approximately 39% of Canada’s population [40]. The province received approximately 40% of all immigrants to Canada in the fourth quarter of 2022 [40]. There are 34 PHUs in Ontario, jointly funded by local governments and the provincial government (Public Health Ontario 2023). Each PHU is responsible for protecting and promoting the health of its residents through the delivery of programs and services in a specific geographic area. PHUs are under the direction of local medical officers of health, who are accountable to boards of health [41, 42].

### Analytic approach

We were guided by a critical realist ontology, which considers the existence of a reality, even if it can only be grasped through the researcher’s interpretation of the perspectives and discourses of research participants, who are actors in the experiential context [43, 44]. Language is conceptualized as a channel for accessing information that reflects the contextual reality of the actors [45]. To focus on the meanings and significance attributed by participants, we adopted an experiential epistemological orientation to data interpretation. The adoption of this approach means that this analysis is not an attempt to explain the social construction of the partnership between FBOs and PHUs, but rather an examination of participants’ subjectivity about the construction of the partnership [46]. An experiential orientation was most appropriate because it allows us to prioritize the PHU respondents’ own accounts of their attitudes and opinions regarding their engagement with FBOs, without attempting to analyze the sociocultural factors that underpinned the development of their attitudes and opinions.

#### Data collection

A total of 34 PHUs were invited to participate, and 18 PHUs agreed to be interviewed. Interviews were conducted via Zoom with one to three staff members from each PHU involved in COVID-19 vaccine implementation (i.e., vaccine managers, program managers, and medical officers of health). A total of 19 in-depth interviews (two with one PHU) were conducted, each lasting between 60 and 80 min. A semi-structured interview guide was used. This provided flexibility to explore participants’ perceptions, experiences, and meanings in depth, while capturing contextualized data and allowing for new lines of inquiry. The guide covered three main topics: (1) experiences of partnering with faith-based organizations; (2) success factors for vaccine trust-building interventions in the context of partnering with faith-based organizations; (3) barriers or challenges to implementing vaccine uptake interventions with faith-based organizations. Interviews were conducted between August and November 2022. Interviews were conducted by three members of the research team (MS, DB, AK). MS conducted 15 interviews, DB conducted 3 interviews and observed 12 interviews, and AK served as an observer for 6 interviews. The team also collected documents about the PHU-FBO collaboration from the interviewees when available. This project received ethics approval from the University of Toronto (#42490).

#### Data analysis

We adopted a primarily inductive approach to analysis, an approach that privileges the meaning of the data from the discourse of the participants rather than from a predetermined theory or conceptual framework [47]. Thematic analysis (TA) was used to generate the coding framework [48]. Specifically, we used an experiential thematic analysis that focuses on exploring the reality of participants’ contextual experiences, perspectives, and behaviors [49]. This method is a kind of midpoint between coding reliability [50, 51] and the reflexive approach to TA (Braun et al. 2022). We implemented six steps: (1) familiarization with the data, (2) systematic coding of the data and development of a codebook, (3) development of an initial theme and consolidation of the codebook, (4) non-rigid coding guided by the codebook, (5) refinement of the codebook and development of the main theme, (6) report writing (see Appendix for details of the analysis process). *Data familiarizatio*n by two team members (KK and AK) involved multiple reads of the raw data, which were then transferred to NVivo 12 for coding. *Systematic data coding and codebook development*: KK and AK first carried out an initial open individual coding of 4 interviews, selected based on PHU size, resource availability, and varying experiences in partnering with FBOs. KK and AK conducted integral coding of each interview based on the interviewees’ discourse [47]. This stage enabled familiarization with the data and the generation of two initial descriptive codebooks. *Systematic data coding and codebook development*: KK and AK first conducted open individual coding of four interviews selected based on three criteria: size of public health unit, resource availability, and experience partnering with FBOs. Each interview was fully coded [47] by KK and AK. *Initial theme development and codebook consolidation*: Following feedback from the core research team, KK and AK merged the two codebooks to create a hierarchically structured version of the initial themes (themes and sub-themes), which was reviewed by ED. This codebook was used (by KK and AK) to code a fifth interview and to review the first four coded interviews. The team completed the codebook consolidation process after three iterations, clarifying the conceptualizations of themes and subthemes with the help of ED, SF, SA, and AA. *Non-rigid coding guided by the codebook* consisted of systematic coding (KK) of the remaining 15 interviews, with emphasis on the semantic and manifest content of the discourse [45]. *The codebook refinement and main theme development* stage involved redefining and refining the initial themes and sub-themes based on our analysis, i.e. identifying the essence of each theme and sub-theme [48]. This made it possible to reorganize (move some content, merge others) to produce a coherent narrative describing the factors that facilitate and hinder commitment to the implementation of vaccine confidence interventions, as well as the challenges associated with working with FBOs from the perspective of PHUs.

## Results

The following results highlight the factors that facilitated and those that hindered the engagement processes during the implementation of interventions aimed at building confidence in COVID-19 vaccines. Our findings describing the processes of engagement are reported elsewhere [52].

### Factors facilitating engagement in the context of vaccine promotion interventions

PHUs have identified two categories of factors that facilitate engagement with FBOs in the implementation of vaccine promotion interventions: (i) factors that facilitate engagement during the planning stage of interventions, (ii) factors that facilitate engagement during the implementation processes of vaccine promotion activities. Below, we present the facilitators of the engagement process during the planning of vaccine promotion activities.

#### Success factors contributing to planning

Internal preparation and planning consisted of moments of reflection between PHUs and FBOs to make choices based on population needs, design of vaccine roll-out or awareness raising strategies and choice of vaccination sites. PHUs outlined four success factors: (i) use of data, (ii) internal collaborative approach, (iii) receptivity and openness to honest discussion with FBOs; and (iv) previous experience working with community and FBOs.

##### PHUs’ use of data

The use of different data sources (e.g., socio-demographics, vaccine uptake) during the planning phase made it possible to target the communities that needed to be reached. Respondents considered this a factor in the success of planning equitable interventions because it made it possible to identify where to intervene.

*“So*,* we had a repository of quite a bit of information so that we could drill into certain FSAs or areas or priority populations that we were seeing emerging through our own data to really build and extend our collaborations and partnerships to understand where we had our own blind spots in terms of where we had connections and where we did not. And then actively reaching out to those populations or those individuals or organizations”*. (Interview_ 9).

This quote illustrates that the use of multiple data sources enabled the PHUs to identify their connectivity gaps in priority areas, leading them to initiate actions to get closer to the population.

##### Internal collaborative approach

PHU’s collaborative approach to internal organization and resource management has enabled them to make effective decisions about the use of human and financial resources, and to carry out internal restructuring to reduce the human resources deficit and meet their partner’s needs. Internal collaboration has thus been a success factor for planning overall due to the enriched nature of knowledge and experience.

PHUs engaged in learning from other PHUs to prepare better and anticipate the actions they needed to take, which was very useful, according to some respondents. *“We weren’t at the center of the pandemic to begin with*,* so we were able to observe what was happening in other communities and anticipate*,* you know*,* okay*,* it’s probably going to happen here too*.” (Interview_10).

##### Receptivity and openness towards a co-design approach

Many PHU respondents shared that receptivity and openness to having honest discussions and learn from FBOs helped maintain and strengthen existing partnerships and/or build trust to form new partnerships. This included engaging in open dialogue with FBOs and faith leaders to understand their communities’ lived experiences impacted by colonization, structural racism, and oppression. Some PHUs were also prepared to support FBOs in whatever ways they wanted to be supported. They did not position themselves as providers of knowledge, but rather active listeners and seekers of advice, thus utilizing a “co-creation” approach. For most respondents, this approach helped establish or maintain trust and made it easier for FBOs to commit to working with PHUs because they had a voice and were listened to by PHUs. For the respondents, the measure of trust is the relationship, the commitment, and the ongoing communication with FBOs such that FBOs get back in touch to ask questions.

*“And to me that’s a bit more*,* even more important … than the actual vaccine at that point. Some of these things*,* especially with generational poverty*,* colonization*,* extended trauma*,* these things are not going to turn around*,* in a day or a month or even a year. So*,* we’ve had great engagement with people who aren’t vaccinated but we’ve come a long way with them. Coming back for information*,* which means they trust*,* or they’re interested or they’re debating. So*,* I think these are*,* for me*,* the indicators… the trust building the relationship*,* the engagement*,* the back and forth. And also*,* but we didn’t position ourselves as the givers of knowledge and information resources*,* but really equally the listeners and the seeking guidance and our whole approach is co-creating”. (*Interview_8)

During the planning phase, openness and honesty meant more co-creation. It was about being open and getting to know the FBOs better and having an honest discussion about the knowledge gaps and the need to learn more about their needs; being receptive in the sense of accepting that certain PHU options may be rejected by the FBOs. This makes it possible to plan activities that are acceptable to the partners.

##### Pre-exiting relationships religious communities and FBOs

Pre-existing relationships between FBOs and PHUs prior to COVID-19 was considered by several respondents as a key success factor in planning vaccine interventions. PHUs relied on their knowledge of these communities, while taking care not to lose this trust by respecting their values and beliefs. Some hesitant communities needed a longer period to decide before deploying their vaccination strategies in these communities.

*“And within their communities*,* it really is community decision making. They do have bishops and leaders that they vote on. You know*,* there’s a lot of community-based decision making. You’re very unlikely to have somebody from the old order community (religious community) participate for instance*,* on an Ontario health family health team*,* as an advisory person*,* like you might have an indigenous representative. So*,* you can’t do it that way. the process is very slow*,* they might come back*,* one year after the first contact and say*,* “we have these additional questions”. They’re very (meticulous) …there’s a lot of discerning and discussion and review and consideration given to all these community decisions and to letting everybody be heard”. (*Interview_5).

This prior knowledge of religious communities was an asset to PHUs, since they were already conscious of the anticipated challenges and collaborative actions to maintain existing trust.

*“We knew we would have those same type of conversations that would be required with COVID vaccine*,* and expected it even more*,* because this was a brand-new vaccine as well. So*,* I*,* I don’t think there was… barriers*,* we knew they wouldn’t want to travel. If we wanted to make access close to their homes because of their transportation needs. We wanted to give them as much information*,* we knew it was gonna take time. We didn’t expect tremendously high rates initially*,* or even in the early months of the vaccine campaign. So*,* I don’t think we experienced anything that we weren’t expecting because we knew the communities”.* (Interview_7)

PHUs tried to retain the same staff who were already known and accepted by vaccine hesitant communities and who know how to interact with discretion. In the case of certain historically hesitant FBOs, the previous partnership enabled honest dialogue on what they are willing to undertake with the PHU.

*“One of the partners in this path forward has always been the radio station as well as the Mennonite community services group. And what these leaders have done with us is partnered over the years to create clear boundaries and clear expectations of what they’re willing to do and what they’re not willing to do. We’re fortunate that their leadership is progressive of saying*,* “you can put this information in this format*,* and we’ll put it on the radio station”*,* and that’s translated information. Or hard lines of*,* “yes*,* we will support that message or no*,* we won’t”.* (Interview_12)

#### Facilitators for successful collaborative vaccine program deployment

Five factors facilitated the collaboration for the implementation of vaccine promotion activities: (i) ongoing relations with FBOs and communities made structurally vulnerable based on respect of different beliefs and opinions on the vaccines; (ii) partnering with FBO leaders and communities to design the vaccination strategy and deliver vaccines; (iii) placing vaccine clinics in trusted areas within targeted communities; (iv) adapting the communication strategy to community characteristics and needs; and (v) FBOs’ openness to collaboration.

##### Respect of different beliefs and opinions on the vaccines

Nurturing sustainable relationships based on respect for different beliefs and opinions about vaccines facilitates the implementation of interventions to strengthen confidence in COVID-19 vaccines. Respondents said they avoided imposing their beliefs and implemented the vaccination strategies chosen by the FBOs in the way that was most convenient for them.

*“We know that they don’t want to have the vaccine*,* to have vaccine clinics. And we know that they have some primary care doctors who they can reach out to in the clinic to vaccinate kids and to get vaccinated. So*,* we were supplying this doctor with vaccines. So that it’ll be accessible to them and is not really like culturally disclosed so that they won’t have these barriers of being against what they believe in*,* in the collective perception of their community*,* because social norms are very influencing on the individual behavior and decisions. So*,* we try to respect the socio norms while we try get them vaccinated in different approach rather than the clinics and the usual visits”*. (Interview_2)

Similarly, PHUs respected the collective beliefs of the FBOs by exercising discretion and creating suitable spaces for those who wanted to receive the vaccine. This also involved using consistent messaging for specific groups (e.g., migrant workers), recognizing that hesitancy is normal, and that the vaccination site is also a place to get answers to questions (which in turn can build trust). The vaccine implementers did not judge people by making certain comments such as “*people have received four doses and you are here for the first one”.* (Interview_12). They gave people time to decide without pressure. In this way, they acknowledged vaccine hesitancy experienced by members of certain faith-based and racialized communities. They did not pressure the community to get vaccinated while showing willingness to support and respond to the communities’ needs.

*When you’re ready, we’re ready. When you’re ready to talk to us, we’ll be here to talk to you and we’ll continue to take baby steps as we always do, and as we’ve always done throughout our immunization journey with many of these communities.*” (Interview_11)

##### Co-leadership approach for vaccines strategies and deliver

PHUs leveraged the support of trusted faith leaders among communities. Partnership with FBOs and religious leaders, and their support in communicating about and distributing vaccines, has strengthened trust in communities for several reasons. Involving PHU staff from the same community as the FBOs facilitated interactions and helped to build trust. Religious leaders in some places of worship helped to raise awareness of COVID-19 and build vaccine confidence. PHUs reported that some religious communities perceived their leaders as having direct links to PHUs (which can inspire confidence in PHUs since the communities trust their religious leaders). Similarly, religious communities perceived that their faith and place of worship were valued (which can reinforce trust in the PHU in COVID-19 vaccines). Respondents mentioned that prior collaboration can reduce mistrust in vaccines and encourage religious communities to ask questions to inform decision-making. Similarly, involvement of faith-based leaders (who are community champions/ambassadors) in vaccine engagement teams can sometimes improve the trust that community members may have in the government and health institutions.

According to PHU respondents, FBOs’ openness to collaboration contributed to the successful deployment of vaccine interventions. FBOs helped recruit open and committed volunteers to provide information and go door-to-door to talk to people before the clinic, helping people book appointments, and answering their questions. The fact that some leaders were allies helped to build trust and deploy the vaccine. Some FBOs’ experience of working and collaborating with public health agencies or other sectors made their tasks easier.

##### Clinic location

The use of mobile clinics and awareness-raising (places of worship, isolated locations) in hesitant and less hesitant communities helped to bring communities closer together and create a climate of serenity. To achieve this, PHUs set up targeted mobile clinics in hard-to-reach communities. Setting up vaccination clinics in places of worship enabled services to be offered in a familiar environment by people whom the communities trust. In the case of more vaccine hesitant communities, such as those living in rural areas, the installation of mobile clinics enabled communities to receive the vaccine without pressure at a location outside of their community. This strategy helped to instill confidence in some people, who finally chose to receive the vaccine. Mobile clinics also enabled people and their families to access and receive vaccines (in cases where, for example, the religious leader had not officially approved a clinic or vaccine). These community clinics offered greater flexibility and allowed PHUs to deploy clinics immediately whenever an FBO called to request vaccine.

##### Communications strategy adapted and sensitive to the needs of the community

PHUs implemented a communications strategy that was adaptable, accessible, and sensitive to community needs. The communications team at some PHUs, with support from FBOs simplified and translated key messages and important documents into different languages to make them accessible. Message content was often developed according to what the community is ready to hear, particularly to a hesitant community and the illiteracy group. For example, for hesitant groups, messages about the need to stay at home to protect others have often been favored over the importance of getting vaccinated.

*“We deal with people with communities like the Anabaptist communities who are mainly rejecting any kind of vaccine*,* not only COVID. But we know that we must deal with that in a harm reduction approach like if you are sick*,* stay at home…*,* we get into good communication with the chiefs of this community*,* the religious leaders. We’re using mainly the harm reduction*,* the hand washing*,* the distancing*,* make sure that people are screening when they come to church. And make sure that when times when we have that closure like at the time when we had like locked down so that they also comply with that. And with good relation*,* they were really complying”.* (Interview_2)

In some cases, door-to-door canvassing was necessary to reach specific communities.

*“We have a school nurse and a baby nurse that are kind of given the portfolio of working with our anabaptist population so they’re key figures. We have a nurse practitioner that’s well immersed within that community … to try and*,* you know*,* get the information to them*,* and answer their questions. So*,* we really utilized our nurses that had the relationships with that population to see if we could certainly offer information to them regarding potentially answering some questions about vaccine hesitancy or vaccinating or not vaccinate. So*,* through them and through their questions and answers with them we were able to*,* you know*,* answer questions that they had”. (*Interview_14)

### Challenges to implementing confidence in vaccines

PHU respondents identified several challenges which hindered PHUs’ engagement with FBOs for the implementation of vaccine confidence interventions. Two categories of challenges were highlighted: those related to the internal capacity of PHUs, and those related to external factors such as other health system actors and working with FBOs.

#### Challenges related to internal PHU capacity

Respondents expressed that their respective PHUs’ pandemic plans were inadequate to fully respond to the COVID-19 pandemic given the unanticipated magnitude and scale at which the highly contagious disease spread, at a time when no vaccines were available. PHUs’ lack of organizational and operational capacity made implementation and partnerships challenging. Some respondents identified that their respective PHUs had low capacity in terms of human resources (expressed by the majority of the PHUs), financial resources, and analytical capacity (expressed by some PHUs); all of which hampered the implementation of interventions to boost vaccine confidence.

##### Low operational capacity of PHU: Insufficient human and financial resources

Collaboration with FBOs required time and patience to build trust. Yet public health interventions were often planned with tight deadlines, giving little time to build collaborations. This situation was reinforced by the lack of infrastructure and community engagement expertise, which limited the ability of PHUs to establish partnerships with FBOs.

The low technical capacity of staff and the limited financial resources of some PHUs prevented them from coordinating actions based on community champions and supporting FBOs, thus not fostering the establishment of trust-building partnerships. Insufficient human resource capacity made it difficult to implement their vaccine confidence initiatives, often due to the difficulty of finding staff to carry out the work. There was also often a lack of in-house expertise, whether within PHUs or in FBOs, to carry out certain activities This challenge was more pronounced in rural areas where there were fewer available local staff with the required skills. Similarly, PHUs found it difficult to retain staff who were overworked and stressed, and to mobilize in-house staff for immunization services. In addition, the redeployment of some nurses (through care and home visits, already integrated into communities that trust them) resulted in shaken confidence, especially in vaccine hesitant communities.

*“In Amish and the Mennonite communities*,* I think one of the bonuses is the fact that we were able to get into some of the families and talk with them one on one. But again*,* with capacity here with our health unit*,* a lot of times our nurses were redeployed. So*,* there was minimal staff and minimal ability to get out there on a regular basis. The redeployment of staff*,* I could see as a barrier as well. And then it does take time to get there”. (*Interview_14).

Insufficient human and financial resources also led PHUs to follow the Ministry of Health’s guidelines and orient their strategies towards more universal interventions (focused on mass immunization), rather than targeted interventions for population made structurally vulnerable and with low confidence in health organizations. The low technical capacity of staff and limited financial resources of some PHUs prevented the coordination of activities with community champions and supporting FBOs. Consequently, this hindered the trust-building process with FBOs.

PHUs were faced with the challenge of navigating between science and misinformation on the Internet from anti-vaccine and faith-based positions; this made communication difficult, especially when certain positions were taken by public figures such as musicians, film actors or religious figures. PHUs also faced the challenge of protecting the privacy of certain faith-based partner organizations from the media, given that media coverage could undermine the trust established with some hesitant FBOs. Some respondents argued that media coverage can undermine the trust being built up with some hesitant FBOs.

##### Weak data and analytical capacity

While in some PHUs the use of data is mentioned as a success factor, this is not the case in PHUs with low analytical capacity. In some instances, there was also a lack of meaningful data or a gap between available data and ability to synthesize and analyze data to create meaning, insight, knowledge to inform decision-making. These PHUs indicated that they often lack the capacity to process data received from the province to support their interventions. For example, it was difficult for some PHUs to use the Ministry of Health’s COVax (central data repository for COVID-19 vaccine data and reporting in Ontario) platform. Some PHU respondents expressed that forward sortation areas (FSAs) did not offer much value when planning their vaccine delivery programs. Low analytical capacity was usually linked to low staff capacity or expertise in this area, even if some PHUs were able to design their own systems and databases to support their planning.

PHU staff also expressed that the provincial data collection system based on the health card was unable to identify certain hard-to-reach population categories (e.g., homeless, undocumented, low-literacy communities), which are often groups excluded from the healthcare system because they do not have health insurance.

*“The ICES (Institute for Clinical Evaluative Sciences) analyses of substance use*,* and homelessness was not accurate. In our opinion*,* they were not accurate because they were based on the use of health services. The challenge with vulnerable populations from a data perspective*,* then*,* is how to identify them using their health card number*,* which has its limitations. They don’t have a health card. Because they weren’t collecting this data*,* they had to rely on other indicators associated with the health card number. If a person had one and used the services of a hospital*,* that would show up because the ICES had used the OHIP number (to) do that analysis. So*,* it depends on how much the person interacts with the healthcare system through the OHIP number”.* (Interview_10)

Some PHUs did not have sufficient knowledge about their communities’ habits, routines, and practices. Unlike FBOs, PHUs produced statistics without really knowing the contextual realities affecting these communities, making it difficult to develop links with these communities and strategize effective interventions to implement within these communities.

*“I think in general*,* we had very good quantitative data*,* but we often lacked information about the community and on the ground. We don’t know the people that FBOs serve*,* or their habits. I think most FBOs*,* and this is probably their greatest strength*,* they know their community very well and have incredible assets to bring that community together. So*,* they can share with us the knowledge that we lack*,* based on our own experiences and position”.* (Interview_9)

##### Ambiguity in the roles of the different actors

PHUs sometimes placed FBO leaders in difficult positions, with roles and responsibilities that were often unclear because they were determined quickly, or in some instances there were sociopolitical differences in perspectives on how to proceed. Respondents mentioned the need to clarify roles, as it was often difficult for the leader to navigate between his or her community and the PHUs and mentioned that some leaders found themselves in an uncomfortable situation when, as part of a partnership, PHUs asked them to implement certain decisions made by the Ministry. Respondents expressed that some leaders preferred certain decisions to be made by PHUs, so as not to have to explain themselves to their communities. According to some respondents, religious leaders wanted to implement the restrictions and masks, but they did not want it to come from them, but rather to have PHUs lead on these matters.

#### Challenges from factors external to PHUs

External contextual factors are those that do not depend directly on internal PHU management. Some of the challenges were linked to the constant evolution of knowledge during the pandemic, which led to recurrent changes impacting health system players (e.g. changes in ministry of health guidelines) while others were related to FBOs.

##### Constant evolution of knowledge about COVID-19

The emergency context of the pandemic and the rapid evolution of knowledge about the disease and the vaccine, posed directive challenges, as the Ministry of Health was building the plane while they were in flight. So, PHUs faced challenges in responding to provincial guidelines issued by the Ministry of Health (MOH) or lack thereof guidance documents were not always ready when PHUs needed them.

*“People often think oh*,* you have a vaccine clinic*,* it’s just a matter of putting the vaccine clinic up and not understanding that no*,* there’s medical directives that need to be written*,* and in order for a medical directive to be written*,* we need to have our guidance document from the ministry outlining it*”. (Interview_1).

Regularly changing provincial guidelines also had a negative impact on PHUs’ response to FBOs, relation to the translation of communications tools; this led to a reworking of communication materials, often resulting in confusion over which versions of document content to consider. These challenges sometimes influenced collaboration between PHUs and FBOs because

“*people didn’t know if they could trust the health departments*,* right? Or if they could trust the ministry”*. (Interview_1)

In addition, the Ministry’s lack of flexibility in prioritizing groups (who to vaccinate first) contributed to worsening inequities already being faced by populations at greater risk of contracting COVID-19.

*“Provincial policy was a big one because we didn’t have too much flexibility at the local level when it came to like priority setting of what groups should get vaccine first. And honestly a lot of the rollout despite local efforts and provincial*,* a lot of health intervention still ended up being inequitable. We did see uptake better in certain groups*,* people that could access it. Healthcare workers that were younger and healthier got it before people that would’ve been in more need. So*,* I think like despite that there were still*,* and continue to be still a lot of inequities. And some of that is like we don’t necessarily have the flexibility at the local level say we’re not implementing the provincial framework kind of thing*,* which made it hard.” (*Interview_18).

Public health restrictions on gatherings also reduced the ability of PHUs to reach certain communities through community groups and gatherings; thus, cutting off a means of contact to hard-to-reach groups.

*“But I think especially for our equity seeking populations. So that’s been a struggle too*,* it’s made it a struggle for us. I know with the vaccine planning*,* we reached out to many*,* many groups. So*,* for example the targeting newcomers*,* we reached out to all of the different groups that were within the city. So*,* looking at supporting people coming from Africa*,* people coming from different areas. Some of the faith-based groups based on that. And we really weren’t successful when it came to vaccine planning because these groups had minimal contact with the people they were supporting. Right. So*,* the lack of congregation really made it difficult for people to be supported and for us to reach them”. (*Interview_13).

The mandates and restrictions that were imposed by the MOH, as well as the enforcement policies, had a negative impact on relationships with certain partners, creating tensions with certain communities, particularly the homeless:

*“the damage done to relationships*,* sometimes even prior to the vaccine program. Some of those enforcement policies made it exceptionally challenging for any movement on policies simply because telling someone that they must do something for many people*,* sort of puts their backup and creates challenges that maybe having had a conversation with them previously might not have…the barrier that exists for us is that relationships were weakened or were stressed throughout the entirety of the pandemic in a variety of phases*,* sometimes related to our own actions and sometimes completely outside of our own control”*. (Interview_3)

Indeed, according to some PHU staff, telling a person what to do infringed on what they thought were their individual rights.

*“I think the biggest barrier that we haven’t discussed that we faced was the challenges around mandates and restrictions and enforcement policies. For our program*,* what we’ve experienced has been the enforcement of policies*,* whether they were internal policies like local policies*,* or provincially driven policies has shifted and oftentimes negatively impacted the relationship that we have with some of the populations or population groups or even individuals. Whereby telling someone that they must do something infringed on what they believed to be their individual rights or what were their individual rights in some respects”. (*Interview_3).

Even when well thought out, MOH decisions have had unintended consequences and sometimes unforeseen impacts at the local level. Lack of understanding of the local sociocultural context can result in a disconnect between local realities and policy and decision making.

##### FBOs’ beliefs, attitudes, and organizational capacity

Organizational and individual factors concerning the leaders may contribute to challenges with the partnership between PHUs and faith-based organizations.

***Desire to maintain credibility*** was identified as a challenge related to faith-based leaders. Indeed, in some hesitant religious communities, a leader who is in favor of COVID-19 vaccines could be perceived as lacking credibility and lose the trust of faith- based communities. Consequently, some faith-based leaders feared losing community support due to a partnership with PHUs. These did not want to lose credibility with their congregations and communities on the vaccine issue. According to some PHU respondents, even if these leaders told them to be in favor of vaccination, they often had a reserved attitude in their speech when addressing their communities. This was due to the fear of losing their congregants, which could lead to the closure of their place of worship and the loss of income.

***Sometimes, community leaders had a different opinion*** from that of public health. In such cases, it was difficult for them to be the messengers of an opinion that they did not share. According to respondents, some religious leaders did not grasp the outstretched hand from PHUs because they had no confidence in the government.

***Organizational challenges related to the FBO that may influence collaboration with PHUs***. The way of life of certain religious communities, often recognized as hesitant, sometimes made collaboration difficult. For example, it was difficult for Mennonite and Amish communities to attend meetings organized by PHUs due to low use of technology, living in isolated areas without access to electricity, television, or radio, travelling on horseback, and speaking only unwritten oral languages.

***Low operational capacity of faith-based and community support organizations*** was also a challenge. FBOs had a limited number of volunteers, who were already working full-time elsewhere, which limited FBOs’ interest in partnering with PHUs. Community organizations, too, had often been preoccupied with the needs of their clients. The implication is that implementing collaborative interventions was often no longer a priority for these organizations.

###### Collective beliefs promoting vaccine hesitancy

Some FBOs had a faith-based perception of the vaccine, ranging from moderate hesitancy to resistance. In cases where religious communities exhibited resistance and skepticism about the vaccine, PHUs decided to focus their efforts on vaccine hesitant groups. In some very hesitant FBOs, it was often difficult to get past the church leader to inform the community. However, according to one of the PHUs, some of the outreach materials delivered stopped at the church leader’s house because he didn’t disseminate the information “, “*it was just you know*,* paper campaigns that we would just give them information. So*,* a lot of times we had an internal group that would review to say*,* is it too much*,* not enough*,* is this what they’re looking for? But oftentimes we would just take with the ministry developed and kind of hand it to a few of the key members.” (*Interview_1).

## Discussion

The findings highlight facilitators and challenges that arose when Ontario’s public health units worked with faith-based groups and religious communities to build trust in vaccines among populations made structurally vulnerable. Although PHUs have regularly referred to populations using terms such as ethnoracial groups and hard-to-reach communities, we use the term structurally vulnerable population, since systemic or structural barriers are the root causes of inequities experienced by these populations [53–55].

This study fills a gap in the research on analyzing the factors that enable or hinder the processes of engaging with FBOs. During the planning phase, the use of data and an internal collaborative approach, pre-existing relationships with communities and FBOs and a co-design approach enabled through a receptiveness and openness to honest discussions with FBOs all facilitated the establishment and maintenance of relationships with diverse communities, rooted in respect for the diverse beliefs and opinions of COVID-19 vaccines. During implementation, an adapted communication strategy sensitive to the needs of religious communities, the support of trusted religious leaders, co-leadership approach through the openness of FBOs to collaboration, all facilitated the establishment of trusting interactions with diverse structurally vulnerable populations to address community concerns about vaccines. On the other hand, the PHUs’ inadequate human and financial resources and analytical capacity, often prevented them from establishing relationships with FBOs. In addition, regular changes in provincial guidelines created tensions in collaboration, negatively impacting trust in the health system.

While most research has analyzed the types of activities and outcomes associated with community engagement, our findings add to the limited research focused on the process of community engagement in health promotion. A systematic review of the literature found that joint intervention planning with community partners prior to implementation was a key factor in facilitating engagement and a greater sense of ownership; this enabled communities to identify their needs through a consultative process and empowered them to participate [56]. A rapid literature review highlights barriers and facilitators to community involvement in the prevention and control of COVID-19 [26]. Like Gilmore, success factors identified by our research include early engagement; an ongoing process that is re-evaluated and modified as necessary; clear roles and responsibilities for all stakeholders; open communication with clear, two-way communication channels; a close link with community-level response efforts; and the use and engagement of pre-existing actors [26]. Best et al. have shown the importance of involving those affected by institutional mistrust in message development and health risk communication in health emergencies such as COVID 19 [57]. Our results also show that an engagement process focused on involving structurally vulnerable populations to take into account their perspectives, needs and expectations and their impact on message development could reduce their distrust of health institutions and improve equity of access to the vaccine.

Consistent with our findings, other research [26, 56] found that factors internal to the public health organization, such as lack of effective staff training (inadequate training and support structures), lack of resources or incentives underestimating the level of coordination and effort required (insufficient time allocated or planned), unclear responsibilities, lack of understanding of the context, are often barriers to implementing community engagement processes.

Brunton and colleague [24] argue that in situations where trust is lacking or there is no history of collaboration, engagement may be difficult to achieve and may have little momentum in terms of sustainability. Our findings showed that existing relationships prior to the pandemic and trust between some PHUs and FBOs, facilitated engagement in vaccine confidence interventions during COVID-19. As shown by Kasstan et al., it may be useful to build on previous public health collaborations with faith-based organizations to implement routine immunization programs during health emergencies such as COVID [58, 59].

On the other hand, the global emergency meant that some PHUs found themselves in new collaborative situations and did not often have enough time to take the process through to formal written agreement. However, Cooper also shows that formal partnerships and commitment also appeared to be key factors in creating a sense of cohesions [56]. Pre-existing relationships contributed to successful engagement between public health and faith-based organizations to increase influenza prevention among hard-to-reach populations in the United States [33].

However, PHUs had established decade-old collaborations with some of these communities, which was leveraged to facilitate trust-based exchanges in vaccine promotion. Our results are also consistent with others [60] that have showed factors such as the imposition of public health guidelines (e.g., containment) or their frequent modification (e.g., masking) can conflict with values such as religious freedom and create attitudes of resistance to their application among hesitant religious communities [61, 62].

Our results show that the multiple changes in COVID-19 guidelines created confusion, sometimes weakening the level of commitment from religious leaders For example, the desire of religious leaders to maintain their credibility in the minds of their congregants, lest they be perceived as leaders who believe more in the health care system at the expense of their faith prescriptions, created a reluctance that jeopardized the development of a partnership, especially in religious communities that depend on membership dues. Moreover, apocalyptic speculations about COVID-19 [63], and the antivaccination skepticism of certain religious leaders often decreased vaccine confidence within certain communities, and hampered vaccination efforts [64]. More generally, committed people of faith may find themselves forced to choose whom to trust - religious leaders or public health experts [62].

The weak organizational capacity of FBOs is also a challenge for partnership building. Santibañez [34] in a study of 24 Association of State and Territorial Health Officials (ASTHO) and four major US cities, found that some agencies had difficulty establishing connections with smaller religious communities (which are not members of FBO coalitions), due to different channels of communication. Like some Ontario PHUs, some ASTHOs have created community engagement departments to integrate community and FBOs into the response to COVID-19 [34].

Our study has several strengths. It sheds light on barriers and enablers to engagement processes between PHUs and FBOs, an area that has received less attention in empirical research. Often, studies focus on the evaluation of results [36] without addressing the factors that contributed to the production of these results, such as the analysis of upstream processes. These results point to the need for contextualized data on facilitators and challenges of participatory engagement processes. By characterizing engagement processes that contributed to increased trust in COVID-19 vaccines among hesitant communities, these findings can better contribute to the effective replication of collaborative interventions. The added value of this research is that it analyzes the community engagement processes with FBOs in the specific context of a global health emergency. Our study has a few limitations; it only reported on the experiences of those involved in public health units; and consequently, FBOs as participants may have provided different perspectives of the engagement process.

The COVID-19 context is characterized by the implementation of public health measures that make it difficult to establish contact (distancing and containment measures as poor access to FBOs due to restrictions on interacting with these communities), and misinformation that reinforces mistrust in public health services. Nevertheless, our results have several implications for strengthening public health practice. Community engagement takes time, sufficient capacity, and dedicated resources to build and sustain trust. Engaging with religious communities is an ongoing process that requires a great deal of time and patience. Even in religious communities that are hesitant or resistant to vaccines, collaboration is necessary. Collaborations developed over time create clear boundaries and clear expectations among partners. When the collaboration exists and is formalized, the players know each other, and PHUs know what is permitted and accepted by the FBOs. Therefore, community engagement processes based on mutual respect for values and beliefs, and that address inequities and structural racism are necessary to reduce mistrust of vaccines and the health system. Prioritizing engagement with FBOs and devoting sufficient human and financial resources over time are necessary to improve vaccine confidence among among populations made structurally vulnerable.

## Conclusion

In Ontario, collaborations between the PHUs and FBOs were initiated to implement interventions aimed at building vaccine confidence among populations made structural vulnerable. The results of this study shed light on the factors facilitating and hindering engagement in the implementation of vaccine confidence interventions, as well as the challenges associated with working with FBOs. Our examination of these experiences during COVID-19 may help shed light on how to collaboratively address ongoing and future public health challenges. Additional research on the engagement processes with historically hesitant communities is also needed to document their experiences and perceptions of engaging with PHUs. This will provide insights from multiple stakeholder perspectives and contribute to policy and practice decisions to improve equity for structurally vulnerable groups.

## Electronic supplementary material

Below is the link to the electronic supplementary material.

Supplementary Material 1

## Data Availability

Full transcripts cannot be shared publicly due to potentially identifying information. The content and words of respondents in interviews could potentially be used to identify individuals. Even anonymization could pose risks to confidentiality. Data are available upon request to the ethics approval by the from the University of Toronto. Tel: +1 416 946-3273; Fax: +1 416 946-5763; ethics.review@utoronto.ca.

## Abbreviations

CBOs: Community-based organizations
FBOs: Faith-based organizations
ICES: Institute for clinical evaluative sciences
PHAs: Public health agencies
PHUs: Public health units
HPCS: Ontario’s high priority communities strategy
